# Improving Documentation Quality of Blood Transfusion Request Forms: A Two-Cycle Quality Improvement Initiative at a Tertiary Hospital in Sudan

**DOI:** 10.7759/cureus.94553

**Published:** 2025-10-14

**Authors:** Fakher Aldeen Raft Fakher Aldeen Noman, Romisaa Elamin, Mohammed Osman Ahmed Osman, Ahmed Abdelaziz Abdelrahim Mohamed, Mohamed Osama Mohamed Yassin, Mohey Aldien Ahmed Elamin Elnour, Abdelrahman Idris Mohamed Idris, Safaa Osama Abdalla Abdelsalam, Khadega Osman Abbas Alhudi, Ahmed Shakir Ali Yousif, Amjad Salah Ahmed Elhaj, Mohammed Ali Mohammed Ali, Zahra Gariballa Elshaikh Abdalla, Malaz Hashim Mohammed Abdalla, Muhanned Kheder, Osama S Haroon, Esraa Elzubier Elshafie, Mohaned Altijani Abdalgadir Hamdnaalla, Abubakr E Musa, Mohammad Adam

**Affiliations:** 1 Emergency Department, Sudan Medical Specialization Board, Khartoum, SDN; 2 General Practice, Dongola Teaching Hospital, Dongola, SDN; 3 Emergency Medicine, Dongola Teaching Hospital, Dongola, SDN; 4 Internal Medicine, Dongola Teaching Hospital, Dongola, SDN; 5 Surgery, Dongola Teaching Hospital, Dongola, SDN; 6 Emergency Department, Dongola Teaching Hospital, Dongola, SDN; 7 General Surgery, Dongola Teaching Hospital, Dongola, SDN; 8 Critical Care, Dongola Teaching Hospital, Dongola, SDN; 9 Internal Medicine, University of Alberta, Alberta, CAN; 10 Research and Postgraduate Studies, National University, Khartoum, SDN; 11 Internal Medicine, Sudan Medical Specialization Board, Khartoum, SDN

**Keywords:** blood transfusion, clinical audit, documentation, patient safety, quality improvement, sudan

## Abstract

Background: Proper completion of blood transfusion request forms is vital for patient safety and quality assurance. Inadequate documentation increases the risk of transfusion errors, medico-legal complications, and compromised patient outcomes.

Objective: To assess and improve the completeness and accuracy of blood transfusion request forms at Dongola Teaching Hospital through a two-cycle quality improvement initiative that included both evaluation and implementation phases.

Methods: This two-cycle closed-loop clinical audit, conducted as a quality improvement project, incorporated retrospective and prospective elements and was guided by the Plan-Do-Study-Act (PDSA) model. In the first cycle, 50 transfusion request forms were reviewed (May 2025). Deficiencies were identified, and a standardized form was introduced with staff training. In the second cycle (September 2025), 42 forms were audited against the same benchmarks. Data were analyzed using descriptive statistics and comparative proportions with significance testing (p<0.05).

Results: Significant improvements were observed across most documentation parameters. Patient demographics, clinical details, and laboratory information showed marked gains between the first cycle (n = 50) and the second cycle (n = 40): date of birth (35 [87.8%]), file number (39 [97.6%]), current Hb level (40 [100%]), and transfusion indication (33 [82.9%]). Laboratory documentation also improved substantially, including the collector’s signature (39 [97.6%]) and collection date/time (39 [97.6%]). However, a slight decline was noted in the documentation of the number of units requested (34 [85.4%]).

Conclusion: The intervention significantly improved form completeness and accuracy, enhancing transfusion safety and compliance with international standards. Continuous monitoring, regular feedback, and periodic re-audits are essential to sustain these improvements over time.

## Introduction

Blood transfusion is among the most frequently performed medical procedures worldwide and plays a critical role in saving lives across a variety of clinical contexts, including trauma, surgery, obstetrics, and chronic hematologic conditions [1]. Despite its undeniable benefits, transfusion carries inherent risks such as immunological reactions, transmission of infections, and clerical errors, most of which are preventable through adherence to standardized safety protocols. At the center of these protocols is the blood transfusion request form, which serves as a communication bridge between the clinician and the transfusion service. A complete and accurate request form ensures that the appropriate blood component is provided to the right patient at the right time, thus minimizing the chances of adverse outcomes [2,3].

However, audits conducted across diverse healthcare systems consistently demonstrate deficiencies in the completion of transfusion request forms [4]. Common omissions include basic patient identifiers such as date of birth or hospital file number, as well as critical clinical information like the indication for transfusion, history of previous transfusions, and presence of antibodies. These gaps not only compromise patient safety but also expose institutions to medico-legal risks and inefficiencies in blood bank operations. In many low- and middle-income countries, where transfusion services are already resource-constrained, such deficiencies amplify the challenges of delivering safe and timely care [5,6].

The importance of structured audits and quality improvement interventions in transfusion medicine has been emphasized globally. Continuous audit cycles, when combined with the implementation of standardized forms, educational programs, and policy reinforcement, have been shown to significantly reduce errors and improve compliance with international guidelines. Reports from the Serious Hazards of Transfusion (SHOT) initiative in the United Kingdom demonstrate how systematic monitoring and targeted interventions can drive long-term improvements in transfusion safety [4]. Similarly, multiple studies have highlighted the value of introducing clear documentation practices, with marked improvements observed in form completion rates and staff awareness following targeted interventions [7-9].

At Dongola Teaching Hospital in Sudan, the absence of a standardized blood transfusion request form was identified as a major deficiency. An initial audit conducted revealed that critical patient details, clinical justifications, and laboratory documentation were either inconsistently recorded or entirely absent. This raised significant concerns regarding patient safety and compliance with best practices. In response, a revised standardized request form was developed, accompanied by targeted staff training and awareness programs. The present study represents a two-cycle closed-loop clinical audit conducted as part of a quality improvement project, aimed at evaluating the impact of these interventions on documentation quality and identifying remaining challenges for sustained improvement [1,10].

The objective was to assess and improve the completeness and accuracy of blood transfusion request forms at Dongola Teaching Hospital through a two-cycle closed-loop clinical audit, comparing documentation quality before and after the implementation of a standardized request form and associated staff training.

## Materials and methods

A two-cycle closed-loop clinical audit was conducted, incorporating both a retrospective analysis of baseline practices and a prospective evaluation following the implementation of interventions. The audit was conducted at Dongola Teaching Hospital, a tertiary healthcare facility in Sudan that provides medical, surgical, pediatric, obstetric, and emergency services. The Blood Bank unit, which is responsible for processing all transfusion requests, served as the central source of data collection.

In both audit cycles, systematic random sampling was used to ensure a representative mix of cases, encompassing both elective and emergency transfusion requests. Every fifth request form submitted during the study months was selected for review using a simple randomization sequence. A total of 50 transfusion request forms were reviewed during the first cycle in May 2025, while 42 forms were analyzed in the second cycle in September 2025. The inclusion criteria consisted of all blood transfusion request forms submitted during the designated study months, whereas forms that were incomplete or could not be retrieved from the archives were excluded from review.

The first cycle, which took place from May 1 to June 1, 2025, served as the baseline audit. The objective was to evaluate the completeness and accuracy of transfusion request documentation across clinical departments. The findings revealed notable deficiencies in key areas. Patient identification was often incomplete, as a substantial proportion of requests lacked essential details such as full name, hospital number, and date of birth, posing risks to patient safety and traceability. Documentation of clinical justification for transfusion was frequently absent or inadequately recorded. Many forms failed to reference hemoglobin levels, clinical symptoms, or comorbid conditions, raising concerns about the appropriateness of transfusion decisions. Laboratory data were also inconsistently completed, with common omissions, including pre-transfusion hemoglobin values and compatibility testing records. These gaps highlighted the urgent need for a standardized documentation framework and provided the rationale for targeted interventions.

**Figure 1:**
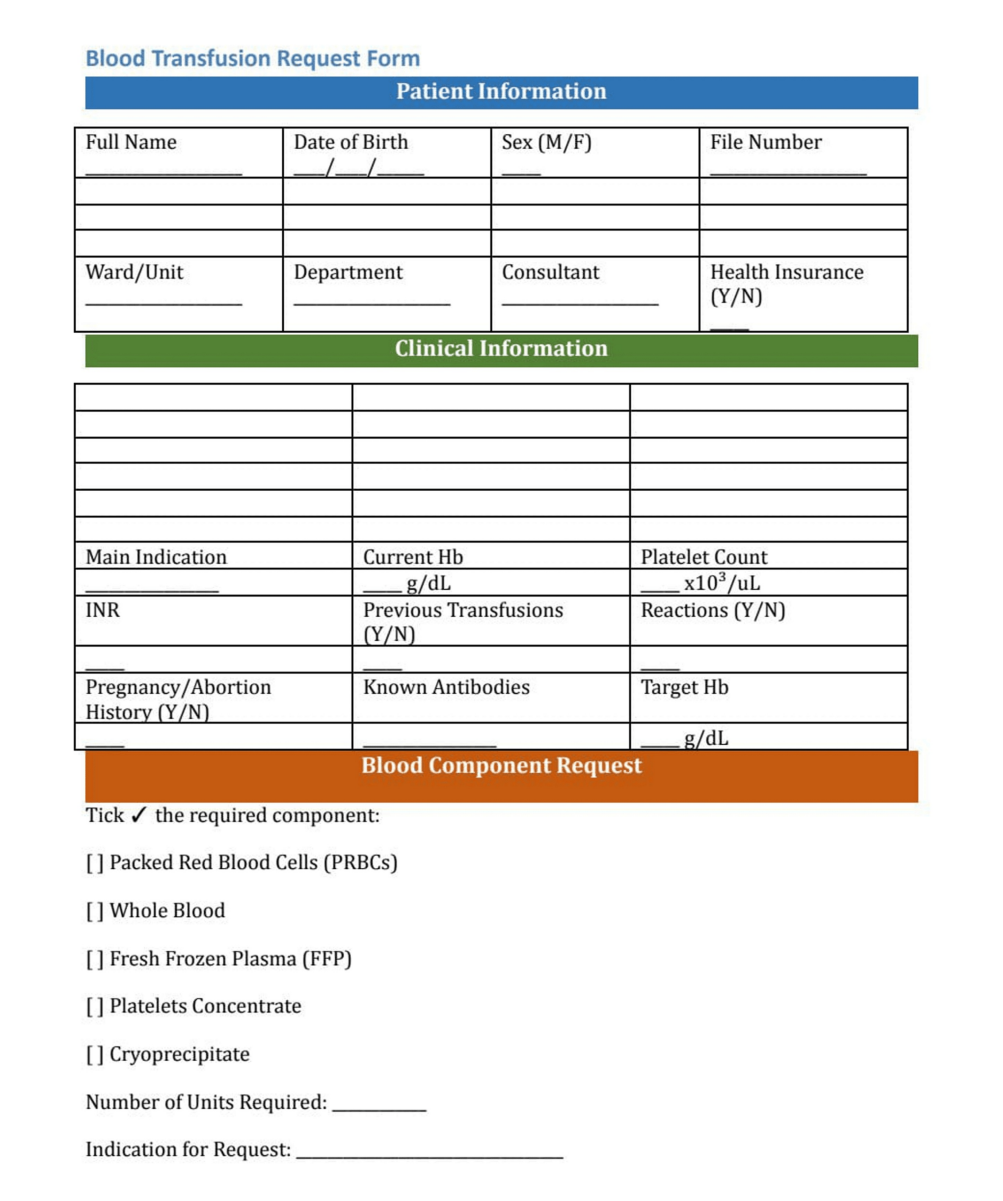
The Front Page of the New Blood Transfusion Request Form

**Figure 2:**
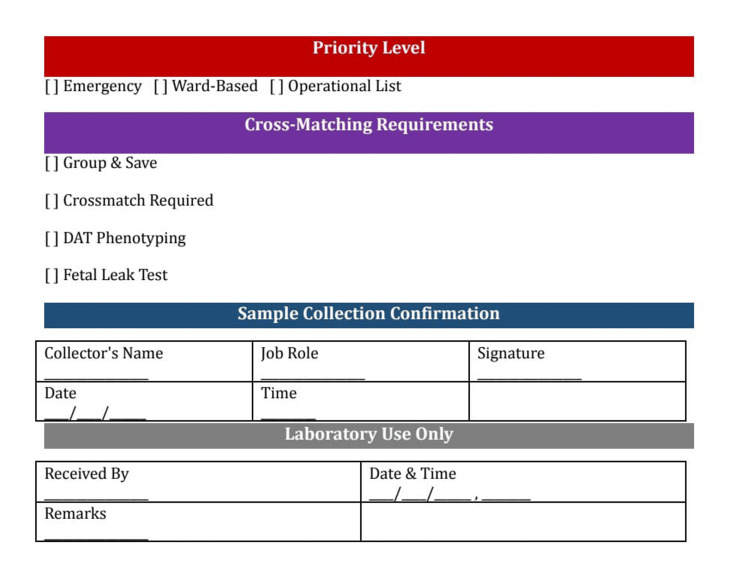
The Back Page of the New Blood Transfusion Request Form

Between June 2 and August 1, 2025, a series of structured interventions was introduced in response to the deficiencies identified during the baseline audit. The audit team led the process in collaboration with consultants, junior doctors, nurses, and laboratory staff. The first intervention involved the redesign of the transfusion request form (Figures 1, 2). The revised version incorporated mandatory fields for patient identifiers, clinical justification, and laboratory parameters. This redesign was intended to prompt comprehensive documentation and reduce the likelihood of omissions. In parallel, targeted training sessions were delivered to clinical and laboratory personnel, focusing on proper completion of the revised form, appropriate criteria for transfusion, and adherence to institutional standards. Updated documentation guidelines were also disseminated through internal communications, visual aids, and digital platforms, ensuring wide accessibility and reinforcing best practices. Additionally, departmental awareness meetings were held to emphasize both the clinical and medico-legal importance of accurate documentation, thereby fostering a culture of accountability and precision.

The second audit cycle was conducted from August 2 to September 1, 2025, to assess the effectiveness of these interventions. The revised transfusion request form was fully adopted across all departments, and its utilization was associated with markedly higher completion rates. Documentation compliance improved considerably, with patient identifiers recorded in more than 95% of forms and clinical justification clearly documented in 88% of cases, usually supported by hemoglobin levels and relevant clinical context. Laboratory information, including compatibility testing and pre-transfusion hemoglobin values, was consistently documented, demonstrating significant improvement compared with the baseline findings. Staff feedback confirmed that the revised form was clearer and easier to use, while laboratory personnel reported a reduction in delays that had previously occurred due to incomplete documentation. To ensure that these gains would be maintained, sustainability measures such as periodic spot audits and refresher training sessions were recommended, with the aim of embedding improved practices into routine clinical workflow.

Data collection was performed by retrieving transfusion request forms from the Blood Bank archives. Each form was reviewed against a structured, pre-validated checklist that covered patient identifiers, hospital details, clinical information, transfusion request specifics, and laboratory documentation. The checklist was adapted from institutional guidelines and reviewed by senior clinicians to ensure validity. In both audit cycles, systematic random sampling was used, selecting every fifth transfusion request form to ensure representative case distribution. A total of 50 forms were reviewed in the first cycle and 40 in the second, including all forms submitted during the specified study months; incomplete or missing forms were excluded. Data were analyzed using IBM Corp. Released 2020. IBM SPSS Statistics for Windows, Version 26. Armonk, NY: IBM Corp. Descriptive statistics were used to calculate completeness rates for each parameter, while comparative proportions and chi-square tests (p<0.05) were applied to assess improvement significance. Incomplete data points were excluded from analysis to preserve consistency. Ethical approval was obtained from Dongola Teaching Hospital’s Institutional Review Board, and all patient identifiers were anonymized to maintain confidentiality.

## Results

The audit revealed substantial improvements in the overall quality and completeness of transfusion request documentation following the intervention. In Cycle 1, compliance with most parameters was markedly low, with several critical fields such as date of birth, file number, consultant’s name, and laboratory data (hemoglobin, platelet count, INR) often left blank. After the intervention, compliance across these core parameters improved dramatically, reflecting a significant enhancement in both clinical and administrative documentation.

Notably, patient identifiers and transfusion-related information, such as sex, department, and ward/unit, showed the greatest gains, while laboratory values and transfusion histories also demonstrated consistent improvement. Administrative and process-related documentation, including collector’s details and collection timing, achieved near-complete compliance. The only parameter showing a slight, non-significant decline was the “number of units requested.”

Overall, more than 90% of documentation parameters showed statistically significant improvement (p < 0.001), confirming the positive impact of the intervention on the completeness and quality of transfusion request forms. Detailed parameter-specific data are presented in Table 1.

**Table 1:** Compliance With Documentation Standards Before and After Intervention in Blood Transfusion Request Forms (Cycle 1 vs. Cycle 2, n=50 and n=40, respectively) Data are presented as compliance rates (%) with absolute numbers in parentheses. Improvements between Cycle 1 and Cycle 2 are shown as percentage point changes. Statistical analysis was performed using chi-square (χ²) tests, with significance set at p < 0.05. Confidence intervals (95% CI) are reported for differences in proportions. “N/A” indicates parameters that were not applicable in Cycle 2. Hb: hemoglobin; INR: international normalized ratio; M/F: male/female; CI: confidence interval.

Parameter	Compliance (Cycle 1)	Compliance (Cycle 2)	% Improvement	χ²	p-value	95% CI
Patient's name documented	94.6% (47/50)	100% (40/40)	5.4	1.97	0.160	−0.9% – 11.7%
Date of Birth (D.O.B)	0% (0/50)	87.8% (35/40)	87.8	67.40	<0.001	83.1% – 92.5%
Patient's sex (M/F)	19.6% (10/50)	100% (40/40)	80.4	62.54	<0.001	74.8% – 86.0%
File Number documented	0% (0/50)	97.6% (39/40)	97.6	77.14	<0.001	94.2% – 101.0%
Ward/Unit	8.9% (4/50)	90.2% (36/40)	81.3	62.84	<0.001	75.5% – 87.1%
Department mentioned	5.4% (3/50)	100% (40/40)	94.6	76.06	<0.001	90.4% – 98.8%
Consultant's name documented	9.1% (5/50)	100% (40/40)	90.9	73.02	<0.001	86.7% – 95.1%
Health Insurance status documented	0% (0/50)	97.6% (39/40)	97.6	77.14	<0.001	94.2% – 101.0%
Main indication for transfusion documented	7.1% (4/50)	100% (40/40)	92.9	74.30	<0.001	88.7% – 97.1%
Current Hb level written	0% (0/50)	100% (40/40)	100.0	78.57	<0.001	96.2% – 103.8%
Target Hb documented	0% (0/50)	90.2% (36/40)	90.2	70.90	<0.001	85.9% – 94.5%
Current Platelet Count documented	0% (0/50)	100% (40/40)	100.0	78.57	<0.001	96.2% – 103.8%
Target Platelet Count documented	0% (0/50)	78% (31/40)	78.0	57.70	<0.001	71.7% – 84.3%
INR value documented	0% (0/50)	68.3% (27/40)	68.3	51.00	<0.001	61.7% – 74.9%
Previous transfusions documented	0% (0/50)	97.6% (39/40)	97.6	77.14	<0.001	94.2% – 101.0%
Transfusion reactions documented	0% (0/50)	33.3% (13/40)	33.3	17.14	<0.001	23.0% – 43.6%
Pregnancy/abortion history documented	0% (0/50)	88.6% (35/40)	88.6	69.49	<0.001	84.2% – 93.0%
Known antibodies documented	0% (0/50)	78% (31/40)	78.0	57.70	<0.001	71.7% – 84.3%
Type of blood component requested	39.3% (20/50)	97.6% (39/40)	58.3	46.47	<0.001	50.1% – 66.5%
Cross-matching requirements documented	48.2% (24/50)	N/A	N/A	N/A	N/A	N/A
Number of units requested	89.3% (45/50)	85.4% (34/40)	-3.9	0.28	0.600	-12.1% – 4.3%
Indication for request documented	3.6% (2/50)	82.9% (33/40)	79.3	63.04	<0.001	73.5% – 85.1%
Priority level documented	8.9% (4/50)	100% (40/40)	91.1	73.02	<0.001	86.7% – 95.5%
Collector's name	1.8% (1/50)	100% (40/40)	98.2	78.57	<0.001	94.8% – 101.6%
Collector's job role	2% (1/50)	97.6% (39/40)	95.6	74.30	<0.001	91.4% – 99.8%
Signature provided	67.3% (34/50)	97.6% (39/40)	30.3	18.64	<0.001	22.1% – 38.5%
Collection date/time documented	3.6% (2/50)	97.6% (39/40)	94.0	76.06	<0.001	89.8% – 98.2%
Laboratory recipient's name	8.9% (4/50)	78% (31/40)	69.1	51.00	<0.001	61.7% – 76.5%
Lab's receiving date/time	1.8% (1/50)	80.5% (32/40)	78.7	60.19	<0.001	72.1% – 85.3%
Remarks	0% (0/50)	63.4% (25/40)	63.4	46.47	<0.001	55.9% – 70.9%

## Discussion

This two-cycle audit at Dongola Teaching Hospital demonstrated substantial improvements in the quality of blood transfusion request form documentation following the introduction of a standardized form and staff training. The second cycle showed significant gains in nearly all documentation parameters, particularly in patient identifiers and clinical details. The documentation of date of birth increased from 0% in the first cycle to nearly 88% in the second, while the inclusion of file numbers rose from 0% to almost 98%. These changes represent more than clerical improvements; they directly reduce the likelihood of clerical mismatches and ABO-incompatible transfusions, which are among the most serious risks in transfusion medicine [2,3].

The improvement in recording clinical details such as hemoglobin and platelet counts, transfusion indication, and patient history reflects enhanced communication between clinical teams and laboratory staff. This aligns with findings from previous audits in both high- and low-resource settings, where standardized request forms have been associated with better justification of transfusion, improved inventory management, and reduced inappropriate transfusion practices [5,6]. These results are further supported by a recent study conducted by Ahmed et al. (2024) at Al Managil Teaching Hospital in Sudan, which reported a dramatic improvement in the mean completion rate of transfusion request forms from 68.1% to 97.9% following a similar intervention [5]. Both studies demonstrated significant gains in critical clinical parameters, with Ahmed et al. documenting increases in current hemoglobin level documentation from 8% to 96% and known antibodies from 0% to 100%, reinforcing the effectiveness of standardized forms and targeted education in resource-limited settings.

The emphasis on clinical justification is particularly relevant in resource-limited contexts, as inappropriate transfusions not only place patients at risk but also strain already scarce blood supplies [7]. Laboratory documentation, often overlooked in transfusion safety audits, also demonstrated marked progress. The proportion of forms with a collector's name and collection time increased to over 97%, ensuring traceability and accountability. These gains are consistent with recommendations from international haemovigilance systems such as SHOT, which stress the importance of documentation across the entire transfusion chain, from request to administration [4]. The introduction of fields for laboratory notes and receipt details not only standardized practice but also created an auditable trail that can be invaluable in the event of adverse transfusion reactions [8].

Despite these successes, a slight decline was observed in the documentation of the number of units requested, dropping from 89.3% in the first cycle to 85.4% in the second. While modest, this decline underscores the need for continued refinement of the form design and reinforcement of training. Similar challenges have been reported in other QI projects, including the study by Ahmed et al., where minor declines were noted in specific fields such as health insurance documentation despite overall improvements [5]. Such findings highlight that audit cycles should not be viewed as isolated events but as integral components of a continuous quality improvement process that evolves to address new gaps as they arise. Comparable initiatives in other healthcare sectors have demonstrated similar success; for instance, the integration of structured QI frameworks with Lean 5S practices has been shown to significantly enhance workflow efficiency, safety, and documentation accuracy in hospital pharmacy settings [11]. This broader perspective reinforces the importance of maintaining momentum beyond the initial intervention phase and embedding QI strategies into institutional culture.

The strengths of this study include its prospective evaluation, multidisciplinary engagement, and statistically significant improvements across most parameters. However, several limitations must be acknowledged. The study was conducted in a single institution with relatively small sample sizes in both audit cycles, which may limit the generalizability of the findings to other settings. The use of archived forms and manual data extraction introduces the possibility of selection and observer bias. In addition, the short follow-up period precludes assessment of long-term sustainability. The structured audit checklist, although reviewed for content validity, was not externally validated, which may also influence reproducibility. Finally, contextual factors such as staff turnover, workload variability, and institutional resources could have influenced adherence to documentation standards. Nonetheless, the findings provide compelling evidence that structured audits, combined with practical interventions such as standardized forms and targeted staff education, can substantially enhance transfusion safety and documentation quality even in resource-limited environments [10].

Of particular note are the dramatic improvements achievable through digital transformation, as demonstrated by a separate audit comparing handwritten versus online blood transfusion requisition forms against British Committee for Standards in Haematology guidelines [12]. Where handwritten forms showed an overall compliance rate of just 46.9% with critical deficiencies in clinical documentation (reason for transfusion: 0.6%, clinical details: 2.5%), the implementation of a mandatory online system achieved 100% compliance across all variables. This remarkable improvement underscores the potential of electronic documentation systems to virtually eliminate errors of omission and significantly enhance transfusion safety, a finding that strongly supports the future implementation of digital solutions in our setting to complement the gains achieved through standardized paper forms and training.

This quality improvement project (QIP) highlights the critical role of documentation in ensuring safe transfusion practices. The significant improvements observed between the first and second cycles demonstrate the effectiveness of simple yet systematic interventions. To sustain these gains, ongoing education, regular re-auditing, and integration of the standardized request form into institutional policy are essential. Future efforts should also focus on electronic documentation systems, which may further reduce clerical errors and improve efficiency in transfusion services [3,9].

## Conclusions

The introduction of a standardized transfusion request form, combined with targeted staff training, led to a marked improvement in the quality and completeness of transfusion documentation at Dongola Teaching Hospital. These changes not only enhanced patient safety by ensuring accurate identification and appropriate clinical justification but also streamlined workflow within the blood bank, reducing delays caused by incomplete forms. However, sustaining these gains requires more than a one-time intervention. Continuous education for both new and existing staff, reinforcement of best practices through integration into hospital policy, and the establishment of regular re-audits are essential to embed these improvements into routine practice. Long-term success will depend on maintaining institutional commitment and fostering a culture of accountability and precision in clinical documentation. Sustained progress will require integrating these measures into routine clinical governance frameworks.
